# Behavioural and psychological symptoms of dementia in patients with Alzheimer’s disease and family caregiver burden: a path analysis

**DOI:** 10.1186/s12877-021-02109-w

**Published:** 2021-03-05

**Authors:** Bokyoung Kim, Gie Ok Noh, Kyunghee Kim

**Affiliations:** 1 Department of Nursing, Catholic Kwandong University, Gangneung, South Korea; 2 Department of Nursing, Konyang University, 158 Gwanjeodong-ro, Seo-gu, Daejeon, 35365 South Korea; 3 Department of Nursing, Chung-Ang University, Seoul, South Korea

**Keywords:** Alzheimer’s disease, Behavioural and psychological symptoms, Burden, Dementia, Family caregivers, Neurology, Path analysis, Symptom cluster

## Abstract

**Background:**

Studies reported the relationship between behavioural and psychological symptoms of dementia (BPSD), cognitive function, caregiver burden, and therapeutic effects. However, the causal relationship between BPSD in community-dwelling patients with Alzheimer’s disease (AD) and caregiver burden is yet to be established. This study aimed to classify BPSD in patients with AD and identify the relationship between BPSD and the factors affecting family caregiver burden.

**Methods:**

Path analysis was conducted at a neurology outpatient clinic of a tertiary general hospital in South Korea. The medical records of 170 patients, aged ≥50 years, diagnosed with or suspected for AD were retrospectively reviewed. We investigated cognitive function (Korean version of the Mini-Mental-State Exam), dementia stages (Korean version of the Expanded Clinical Dementia Rating scale), depression (Short-form Geriatric Depression Scale-Korea), activities of daily living (ADL; Korean version of the Barthel Activities of Daily Living index), instrumental activities of daily living (IADL; Seoul-Instrumental Activities of Daily Living), and BPSD and caregiver burden (Korean Neuropsychiatric Inventory). Considering the characteristic features of BPSD with various symptoms, BPSD was classified using factor analysis. Factor extraction was performed using principal component analysis, followed by Varimax factor rotation.

**Results:**

Mean total BPSD score was 17.66 ± 20.67, and the mean score for family caregiver burden was 9.65 ± 11.12. Symptom cluster-1 (hyperactivity symptoms) included disinhibition, irritability, and agitation/aggression. Symptom cluster-2 (psychosis symptoms) included hallucinations, anxiety, elation/euphoria, delusions, and depression/dysphoria. Symptom cluster-3 (physical behaviour symptoms) included appetite and eating abnormalities, apathy/indifference, aberrant motor behaviour, sleep, and night-time behaviour disturbances. Dementia stages, ADL, and IADL had indirect effects on family caregiver burden through hyperactivity, psychosis, and physical behaviour symptoms, indicating that BPSD exerted a complete mediating effect.

**Conclusions:**

Unlike previous studies, we classified BPSD symptoms into similar symptom clusters to evaluate its effect on caregiver burden, rather than collectively investigating the 12 symptoms of BPSD. As the dementia stage worsens, symptom clusters in BPSD serve as a medium between ADL and IADL degradation and for the increase in caregivers’ burden. The development and implementation of therapeutic, nursing interventions, and policies focusing on dementia stages, ADL, and IADL, delaying and preventing BPSD can alleviate family caregivers’ burden.

## Background

The prevalence of dementia in the elderly in South Korea was reported to be 10.7%, and the number of patients with dementia is estimated to exceed 3 million by 2050 [1]. Alzheimer’s disease (AD) is the most common type of dementia, accounting for 60–70% of all patients with dementia [2].

The prevalence of non-cognitive behavioural and psychological symptoms of dementia (BPSD) in patients with AD is 56–98% in the community and up to 91–96% in hospitals or long-term care facilities [3]. BPSD exacerbates cognitive decline and physical dysfunction in patients with AD [4] and imposes a great burden and stress on the caregivers of patients with dementia [3, 5].

In particular, family caregivers of patients with dementia were found to be associated with social isolation, physical health deterioration, and psychological disorders such as depression. Likewise, these people were also found to experience financial difficulties [6]. Owing to the prolonged disease course, the burden on family caregivers has led to emerging social problems such as conflicts within the family, deterioration of the patient–caregiver relationship, patient abuse, and suicides. Therefore, studies on family caregiver burden are warranted.

Factors related to caregiver burden as reported in previous research are cognitive function [5], stages of dementia [7], depression [7], activities of daily living (ADL) [8], instrumental activities of daily living (IADL) [9], and BPSD [5, 10]. However, it is reported that cognitive function [11], disease progression [12], depression [11], ADL [11], and IADL [13] are related to BPSD. In addition, some studies have reported on the relationships between BPSD, cognitive function, caregiver burden, and therapeutic effects [14]. However, causal relationships between such factors have not yet been established. Furthermore, since caregiver burden and BPSD have been studied with a focus on a single symptom, BPSD expression characteristics have not been fully reflected. As BPSD manifests with the co-occurrence of various symptoms, a trend to investigate BPSD as symptom clusters or groups of related symptoms in a wide range [15] has been established. Previous studies have reported on the classification of the clusters of BPSD symptoms in patients with dementia [15–17]. Garre-Olmo et al. [16] classified BPSD into psychotic, emotional, and behavioural syndromes. In South Korea, a study reported on factor analyses of BPSD in patients with AD living in an institutional setting [18], and another study reported the effects of BPSD in elderly patients with dementia in nursing homes [5]. However, studies regarding the causal relationship between BPSD in community-dwelling patients with AD and caregiver burden have not yet been established. There is also a lack of research that classifies BPSD as symptom clusters to determine how each cluster affects caregiver burden and which clusters have the greatest impact on burden. If we determine which symptom cluster affects caregiver burden and which BPSD makes up these clusters, then the same treatment and nursing approach could be employed for these symptoms [15].

The present study aimed to (i) classify BPSD in patients with AD, (ii) identify the causal relationship between BPSD and the factors affecting family caregiver burden, and (iii) provide empirical data for the development of systematic therapeutic and nursing intervention programmes to address family caregiver burden.

## Methods

### Study design

A path analysis was designed to predict causal factors of BPSD in patients with AD and the corresponding burden of the family caregivers. The study is prepared in accordance with the STROBE guidelines and regulations.

### Use of the conceptual framework

We constructed a framework based on previous reviews to identify the factors affecting burden among the family caregivers of patients with AD. These reviews found that the disease characteristics of patients with AD, such as cognitive function [11], disease progression [12], depression [11], ADL [11], and IADL [13] as perceived by the caregivers, were related to BPSD. However, cognitive function [5], stages of dementia [7], depression [7], ADL [8], IADL [9], and BPSD [5, 10] were related to caregiver burden.

**Fig. 1:**
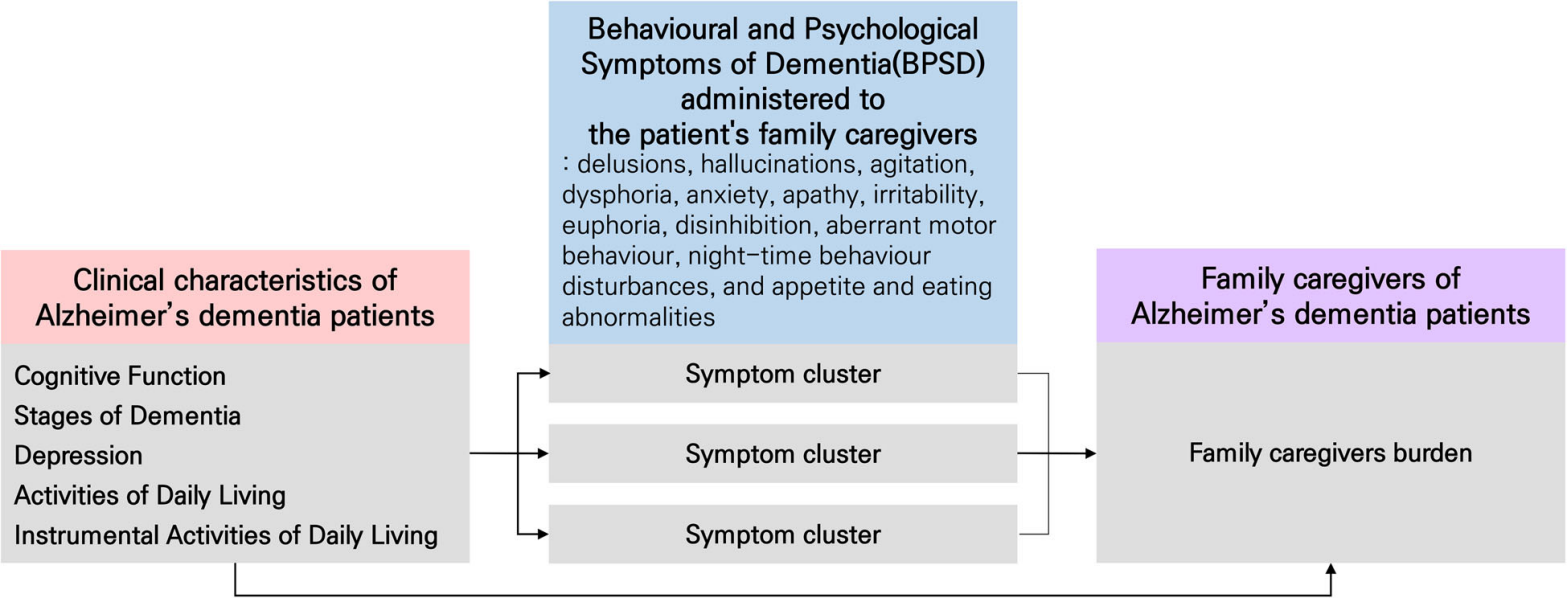
Conceptual framework

**Fig. 2:**
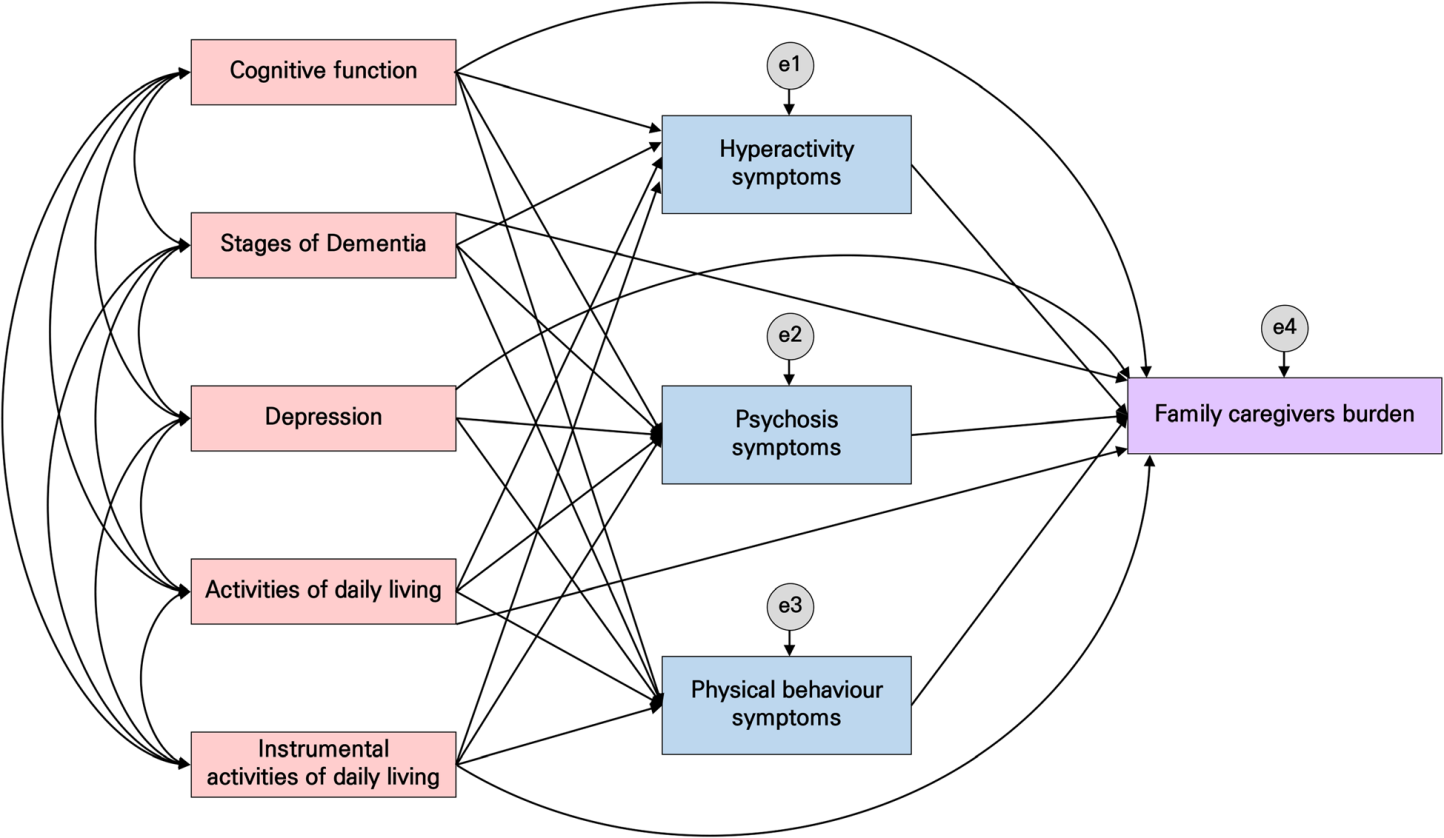
Path diagram of the hypothetical model

Based on these results, cognitive function, stages of dementia, depression, ADL, IADL, and BPSD were selected as the primary influencing factors of family caregiver burden. Figures 1 and 2 show the conceptual framework consisting of the paths in which these factors directly and indirectly, respectively, affect family caregiver burden through BPSD.

### Data collection and ethical considerations

This study used the data retrospectively collected from the medical records of 170 patients with AD who visited the neurology outpatient clinic of a tertiary general hospital in South Korea between January 2011 and February 2016. The study was approved by the Institutional Review Board (IRB) Department of Chung-Ang University Hospital (approval No.: C20160791822) for ethical protection of the participants and for accessing their medical records, including the electronic medical records (EMR) of the respective patients. Government-designated Clinical Research Center for Dementia (CRCD) caregiver questionnaires were completed by the family caregivers. The institutional review board waived the requirement of a written-informed consent from patients and caregivers because the data were historical and had already been collected for diagnostic and therapeutic purposes. The institution also determined that this study was eligible for an exempt status based on the exempt application stating that private data, such as those of patient hospital number, name, and family caregivers’ contacts, will not be recorded. The original CRCD caregiver questionnaires were administered by the staff of the neurology outpatient department and stored in the laboratory of the neurology outpatient department. The EMR of the patients and CRCD caregiver questionnaires were viewed and recorded only in the laboratory to foster patient confidentiality. Serial numbers were assigned to each patient. Software files were stored in encrypted folders on computers with access only to the researcher.

Data on cognitive function, stages of dementia, depression, ADL, IADL, BPSD, and family caregivers’ burden were collected from medical records including patients’ EMR and CRCD caregiver questionnaires. A total of 170 questionnaires were analysed.

### Participants

The participants in this study were patients who (i) were diagnosed with or suspected for AD, (ii) aged ≥50 years, (iii) visited the neurology outpatient clinic of a tertiary hospital in South Korea, and (iv) whose family caregivers completed the CRCD questionnaires. In this study, the classification of AD(diagnosed or suspected) utilised diagnostic criteria of American Psychiatric Association DSM-IV standards, National Institute of Neurological and Communicative Disease and Stroke/Alzheimer’s Disease and Related Disorders Association (NINCDS-ADRDA) [19, 20]. In case there were multiple data for the same patient who visited our institution between January 2011 and February 2016, we used the recent data. The minimum number of samples required for factor analysis is 75–100, wherein an excellent-level criterion is 0.98, and the number of factors is 3 [21]. If the number of samples required for a path analysis is 100 or more, then it is deemed an appropriate sample size [22]. Therefore, the data of 170 subjects were appropriate for the analysis.

### Instruments

#### Cognitive function

Cognitive function identified from the medical records was measured using the Korean version of the Mini-Mental State Examination (K-MMSE), which was translated into Korean by Kang et al. [23] based on the original version by Folstein et al. [24] The K-MMSE consists of a total of 30 items regarding orientation for time, place, registration, attention and calculation, recall, language, and visual construction. Each item is scored as 1 point per factored answer. A lower score indicates more severe cognitive impairment; a score of ≥24 is classified as *non-cognitive impairment*; 18–23, *mild cognitive impairment*; and ≤ 17, *severe cognitive impairment* [25].

#### Stages of dementia

The stages of dementia identified from the medical records were measured using the Korean version of the Expanded Clinical Dementia Rating (CDR) scale, which was translated into Korean by Choi et al. [26] based on the original CDR version by Hughes et al. [27] This scale consists of 6 domains: memory, orientation, judgement and problem-solving, community affairs, home and hobbies, and personal care. This scale is used to classify dementia into 5 stages. The score for the overall dementia stage is determined after comparing the base score for memory with the score for the other items. A score of 0 indicates *non-demented*; 0.5, *very mild dementia*; 1, *mild dementi*a; 2, *moderate dementia*; 3, *severe dementia* [26].

#### Depression

The levels of depression identified from the respective medical records in this study were measured using the Short Form of Geriatric Depression Scale-Korea (SGDS-K), which was modified for the Korean elderly by Cho et al. [28] based on a 15-item excerpt from the 30-item Geriatric Depression Scale developed by Yesavage et al. [29] The SGDS-K is a binary scale consisting of close-ended questions. A score of ≥8 was interpreted as *having depression*.

#### Activities of daily living

The ADL identified through the respective medical records were measured using the Korean version of the Barthel ADL Index (K-BADL), originally reported by Wade and Collin [30] and translated into Korean by Kim et al. [31] The ADL Index consists of 10 items: bowels, bladder, glooming, toilet use, feeding, transfer, mobility, dressing, stairs, and bathing. The total score ranges from 0 to 20 points; a high score indicates an *independent state*, and a score of ≤14 indicates *a state in need of help from others* [30].

#### Instrumental activities of daily living

The IADL identified from the respective medical records in this study was measured using the Seoul-IADL (S-IADL), which was reconstructed by Koo et al. [32] based on the original IADL version reported by Lawton and Brody [33]. The IADL measures more complex functions than the ADL and consists of 15 items regarding the skills and actions required for maintaining a social life. The score for each item ranges from 0 to 3 points. The items included were actions such as using the telephone, shopping, preparing food/cooking, household chores, using the transportation, walking outdoors, taking medication, managing finances, grooming, using household appliances, managing belongings, unlocking and closing the entrance door, maintaining appointments, talking about recent events, and leisure/hobbies activities. A high score indicates *higher levels of dependence*; whereby a score of < 8 is classified as being *independent*, and a score of ≥8 is classified as *needing help* [32].

#### Behavioural and psychological symptoms of dementia and family caregiver burden

The BPSD and family caregiver burden identified through respective medical records in this study were measured using the Korean Neuropsychiatric Inventory (K-NPI), originally proposed by Cummings et al. [34] and Kaufer et al. [35], which was translated and modified into Korean by Choi et al. [36] K-NPI was included in CRCD caregiver questionnaires. The BPSD was scored by monitoring the caregivers’ responses obtained from a self-reporting questionnaire wherein they selected the frequency (4-point scale) and severity (3-point scale) of the symptoms. The frequency and severity scores for each symptom were multiplied, and then the sum of the scores was used to measure the BPSD. A higher score for each symptom indicates a higher severity of the respective symptom, and a higher total score indicates a greater severity of overall BPSD. The caregiver burden for each BPSD item was rated from 0 to 5 points, and a higher total score indicates a higher caregiver burden.

### Statistical analysis

The data were analysed using SPSS 22.0 and AMOS 22.0 programmes. The characteristics of the patients and their family caregivers were analysed using frequency, percentage, mean, and standard deviation. BPSD was classified using factor analysis. Factor extraction was performed using principal component analysis, followed by Varimax factor rotation. Before performing factor analysis, suitability of the data was tested using the Kaiser–Meyer–Olkin (KMO) and Bartlett’s tests. The internal reliability of the classified BPSD symptoms was measured using Cronbach’s α. The correlation between the measured variables was determined using the Pearson’s correlation coefficient.

The hypothetical model was tested using the structural equation model of analysis. The goodness-of-fit of the model was tested using χ^2^/df, Goodness- of Fit Index (GFI), Adjusted Goodness-of Fit Index (AGFI), Comparative Fit Index (CFI), Normed Fit Index (NFI), Incremental Fit Index (IFI), Standardized Root Mean Square Residual (SRMR), and Root Mean Square Error of Approximation (RMSEA) [37]. The significance of the effects of the study model was tested using the bootstrap method.

## Results

### Characteristics of the patients and family caregivers

The mean total BPSD score was 17.68 ± 20.67, and the mean score for family caregiver burden was 9.65 ± 11.12. The characteristics of patients and family caregivers are shown in Table 1.

**Table 1:** Characteristics of the family patients and family caregivers (*N* = 170)

Characteristics				n (%) or Mean ± SD
Patients	General	Sex	Male	57 (33.5)
			Female	113 (66.5)
		Age (years)	50–74	66 (38.8)
			≥75	104 (61.2)
				76.5 ± 8.0
		Level of education	Never educated	62 (36.5)
			Primary–junior high school	60 (35.3)
			High school or above	48 (28.2)
		Smoking	Yes	66 (38.8)
			No	104 (61.2)
		Drinking	Yes	60 (35.3)
			No	110 (64.7)
	Clinical	Cognitive function	≥24 (no cognitive impairment)	98 (57.6)
			18–23 (mild cognitive impairment)	54 (31.8)
			≤17 (severe cognitive impairment)	18 (10.6)
		Stages of dementia	0.5 (very mild)	53 (31.2)
			1 (mild)	102 (60.0)
			2 (moderate)	15 (8.8)
		Depression	< 8 (no depressive symptom)	109 (64.1)
			≥8 (having depressive symptoms)	61 (35.9)
		Activities of daily living	≤14 (help needed)	21 (12.4)
			> 14 (help not needed)	149 (87.6)
		Instrumental activities of daily living	< 8 (help not needed)	30 (17.6)
			≥8 (help needed)	140 (82.4)
		BPSD		17.68 ± 20.67
Family caregivers	General	Sex	Male	65 (38.2)
			Female	105 (61.8)
		Age (years)	20–49	58 (34.1)
			50–64	69 (40.6)
			65–74	26 (15.3)
			≥75	17 (10.0)
				55.4 ± 13.4
		Relationship with the patient	Spouse	48 (28.2)
			Son/daughter	98 (57.6)
			Other	24 (14.1)
		Living with the patient	Yes	108 (63.5)
			No	62 (36.5)
		Burden		9.65 ± 11.12

*BPSD* Behavioural Psychological Symptoms of Dementia

### Classification and type of BPSD symptom clusters

The KMO measure is a value that verifies the correlation matrix to determine the goodness-of-fit for the sample size of all the variables. The KMO measure in this study was 0.83, which was higher than the standard value of 0.50, indicating an appropriate sample size. In addition, Bartlett’s test of sphericity showed that statistical significance was less than 0.001, thereby confirming the goodness-of-fit of the model.

Using a factor analysis based on the severity of 12 symptoms of BPSD, three factors were extracted. The variance for Factor 1, Factor 2, and Factor 3 was 2.85, 2.62, and 2.02, respectively. The explanatory power of Factor 1, Factor 2, and Factor 3 was 23.7, 21.8, and 16.8%, respectively. The total explanatory power of the three factors was 62.3% of the total variance.

Symptom cluster 1 (Factor 1) described *hyperactivity symptoms*, which included disinhibition, irritability, and agitation/aggression. Symptom cluster 2 (Factor 2) described *psychosis symptoms*, which included hallucinations, anxiety, elation/euphoria, delusions, and depression/dysphoria. Symptom cluster 3 (Factor 3) described *physical behaviour symptoms*, which included appetite and eating abnormalities, apathy/indifference, aberrant motor behaviour, sleep, and night-time behaviour disturbances. Cronbach’s α was 0.85 for hyperactivity symptoms, 0.77 for psychosis symptoms, and 0.68 for physical behaviour symptoms (Table 2).

**Table 2:** Factor analysis and classification of BPSD symptom clusters (*N* = 170)

Cluster	Symptom	Factor 1	Factor 2	Factor 3
Symptom cluster 1 Hyperactivity symptoms	Disinhibition	0.87		
	Irritability	0.84		
	Agitation/aggression	0.75		
Symptom cluster 2 Psychosis symptoms	Hallucinations		0.71	
	Anxiety		0.70	
	Elation/euphoria		0.70	
	Delusions		0.64	
	Depression/dysphoria		0.54	
Symptom cluster 3 Physical behaviour symptoms	Appetite and eating abnormalities			0.84
	Apathy/indifference			0.65
	Aberrant motor behaviour			0.59
	Sleep and night-time behaviour disturbances			0.44
Cronbach’s α		0.85	0.77	0.68
Eigen values		2.85	2.62	2.02
Explained variance (%)		23.7	21.8	16.8
Cumulative variance (%)		23.7	45.5	62.3

### Correlation between clinical characteristics and BPSD in patients and family caregiver burden

Family caregiver burden is positively correlated with the stages of dementia, IADL, hyperactivity symptoms, psychosis symptoms, and physical behaviour symptoms and negatively correlated with cognitive function and ADL (Table 3).

**Table 3:** Correlation between clinical characteristics and BPSD in patients and family caregiver burden (*N* = 170)

	1	2	3	4	5	6	7	8	9
1. Cognitive function	1								
2. Stages of dementia	−0.62*	1							
3. Depression	−0.12	0.05	1						
4. Activities of daily living	0.22*	−0.35*	−0.07	1					
5. Instrumental activities of daily living	−0.35*	0.59*	−0.04	− 0.65*	1				
6. Hyperactivity symptoms	−0.24*	0.32*	− 0.16*	− 0.36*	0.43*	1			
7. Psychosis symptoms	−0.32*	0.43*	0.01	−0.25*	0.44*	0.62*	1		
8. Physical behaviour symptoms	−0.29*	0.40*	0.01	−0.35*	0.41*	0.57*	0.57*	1	
9. Family caregiver’s burden	−0.29*	0.39*	−0.02	− 0.35*	0.49*	0.74*	0.81*	0.71*	1

*p < .05

*BPSD* Behavioural Psychological Symptoms of Dementia

### Testing the hypothetical and modified models

**Fig. 3:**
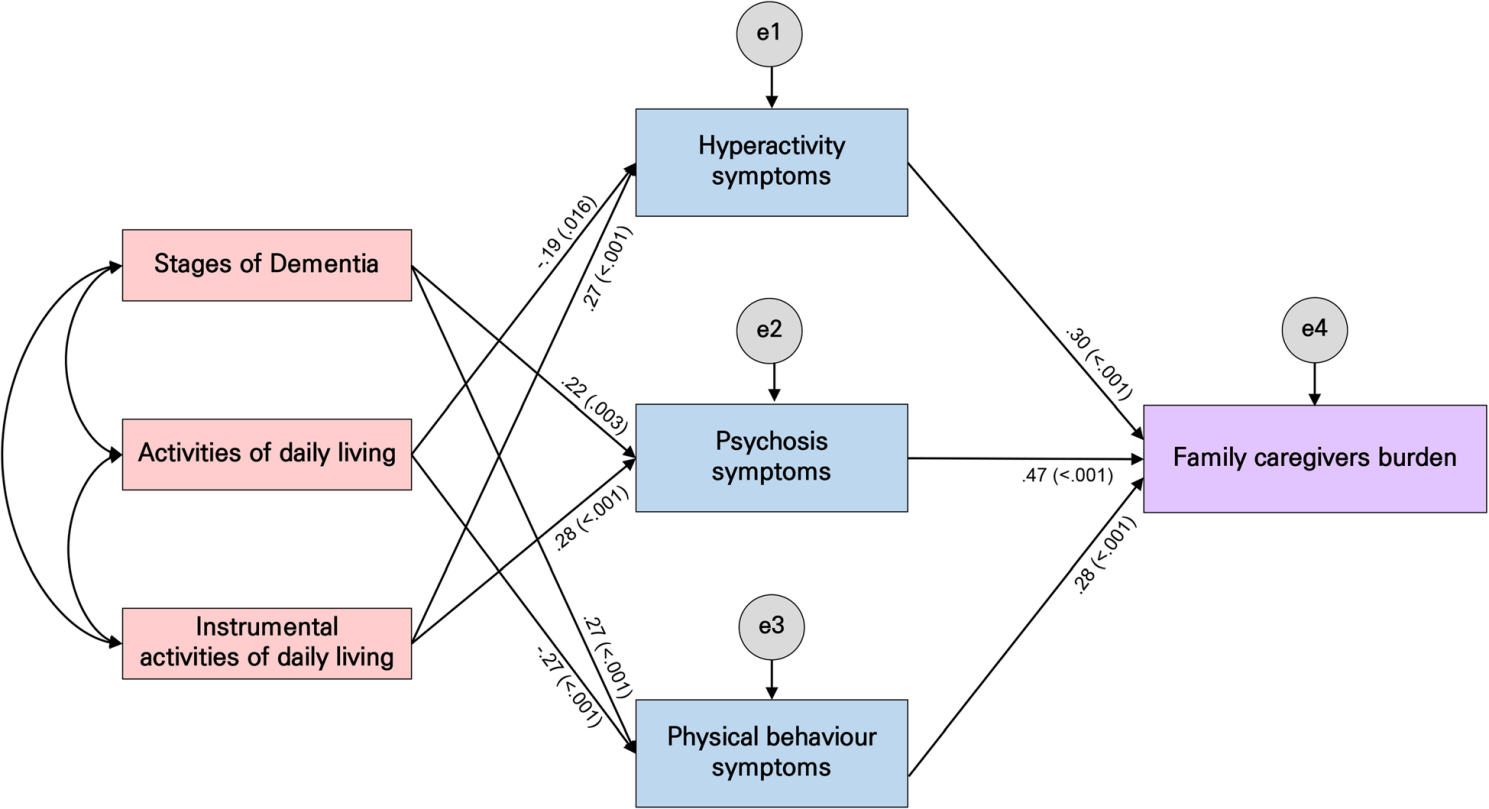
Path diagram of the modified model

While the results of testing the goodness-of-fit of the hypothetical model (Fig. 2) showed that GFI = 0.99, CFI = 0.99, IFI = 0.99, and SRMR = 0.36 met the goodness-of-fit criteria, the results showed that χ^2^/df = 6.56, AGFI = 0.62, and RMSEA = 0.18, which indicated poor fit. To improve reliability, the model was modified, removing insignificant paths based on the significance level of 0.05 while simultaneously verifying the model fit and modification indices (Fig. 3). Successively, the goodness-of-fit indices of the modified model were χ^2^/df = 1.46, GFI = 0.99, AGFI = 0.93, CFI = 0.99, NFI = .099, IFI = 0.99, SRMR = 0.03, and RMSEA = 0.05, which indicated a good fit.

The model indicated that nine paths were significant (Table 4, Fig. 3). The results of analysing the direct, indirect, and total effects of the modified model (Table 4) showed that ADL (γ = − 0.186, *p* = 0.016) and IADL (γ = 0.273, *p* < 0.001) had significant direct and total effects on hyperactivity symptoms; stages of dementia (γ = 0.22, *p* = 0.003) and IADL (γ = 0.28, *p* < 0.001) had significant direct and total effects on psychosis symptoms, while the stages of dementia (γ = 0.27, *p <* 0.001) and ADL (γ = − 0.27, *p <* 0.001) had significant direct and total effects on physical behavioural symptoms.

**Table 4:** Direct, indirect, and total effects of the modified model (*N* = 170)

Endogenous variables	Exogenous variables	C.R.	(*p*)	SMC	Direct effect		Indirect effect		Total effect	
					SE (*p*)		SE (*p*)		SE (*p*)	
Hyperactivity symptoms	Activities of daily living	−2.41	(0.016)	0.18	−0.19	(0.016)			−0.19	(0.016)
	Instrumental activities of daily living	3.34	(<.001)		0.27	(<.001)			0.27	(<.001)
Psychosis symptoms	Stages of Dementia	3.02	(0.003)	0.20	0.22	(0.003)			0.22	(0.003)
	Instrumental activities of daily living	3.71	(<.001)		0.28	(<.001)			0.28	(<.001)
Physical behaviour symptoms	Stages of Dementia	4.00	(<.001)	0.19	0.27	(<.001)			0.27	(<.001)
	Activities of daily living	−3.96	(<.001)		−0.27	(<.001)			−0.27	(<.001)
Family caregiver’s burden	Hyperactivity symptom	6.19	(<.001)	0.78	0.30	(<.001)			0.30	(<.001)
	Psychosis symptom	9.69	(<.001)		0.47	(<.001)			0.47	(<.001)
	Physical behaviour symptom	5.96	(<.001)		0.28	(<.001)			0.28	(<.001)
	Stages of Dementia						0.18	(0.002)	0.18	(0.002)
	Activities of daily living						−0.13	(0.005)	−0.13	(0.005)
	Instrumental activities of daily living						0.21	(0.005)	0.21	(0.005)

*C.R.* Critical Ratio, *SMC* Squared Multiple Correlation, *SE* Standard Estimates

Factors affecting family caregiver burden showed direct and total effects in the order of psychosis (γ = 0.47, *p <* 0.001), hyperactivity (γ = 0.30, *p <* 0.001), and physical behaviour (γ = 0.28, *p <* 0.001). Stages of dementia, ADL, and IADL had significant indirect and total effects, and stages of dementia (γ = 0.18, *p* = 0.002) and IADL (γ = 0.21, *p <* 0.001) had significant positive indirect and total effects. However, ADL (γ = − 0.13, *p* = 0.005) had significant negative indirect and total effects. Therefore, stages of dementia, ADL, and IADL had indirect effects on family caregiver burden through hyperactivity, psychosis, and physical behaviour symptoms, indicating that BPSD had a complete mediating effect.

## Discussion

Since BPSD is the most difficult problem to deal with for family caregivers, who are the primary caregiver for the elderly with dementia, figuring out how BPSD affects the family caregivers’ burden is critical in that it can provide not only the well-being of the family caregivers but also further basic data that can contribute to improving the quality of life (QOL) of AD patients. Our study focuses on classifying BPSD as a symptom cluster and, subsequently, linking these factors that affect BPSD to each cluster, which differentiates this study from previous studies.

Mean scores for BPSD were higher than those for family caregiver burden in patients with AD. These scores of our study were higher than those reported in a study [38] of patients with AD living in veterans’ homes in Taiwan using the same tool. However, these scores were lower than those reported in a study of outpatients with dementia in Portugal [39]. The reason that the scores for BPSD and caregiver burden were lower in the study by Hsu et al. [38] than in our study could be because the subjects of the former were living in veterans’ homes and their caregivers were nurses, whereas the patients of this study were diagnosed with AD at the outpatient clinic at a tertiary general hospital and their caregivers were family members. It is also thought that the subjects of the study conducted by Melo et al. [39] had various types of dementia, including AD, frontotemporal dementia, and vascular dementia. The findings of this study indicate that a higher BPSD is associated with a higher family caregiver burden. However, because there is a difference in the subjects, their residence, and the characteristics of caregivers between this study and previous studies, it is thought that there may be a variable difference in the levels of BPSD and caregiver burden. Nevertheless, 71.8% of the subjects were under-educated people, and it is necessary to check the impact of this education level. In the study of García-Alberca et al. [40], years of education, one of the AD patient general characteristics showed correlation with a range of depression in the tests of measurements taken to determine caregivers’ burden. However, another research [41], which showed no correlation between years of education and caregiver burden, the other [42] showed a faster cognitive decline in AD patients with higher education levels. Further studies with repetition of the analyses will be needed to determine whether or not patient’s cognition and caregiver burden are related to the patient’s education level.

The results of the factor analysis of 12 symptoms of BPSD showed that BPSD can be classified into three symptom clusters: *hyperactivity, psychosis,* and *physical behaviour symptom clusters*. *Hyperactivity symptoms* consisted of disinhibition, irritability, and agitation/aggression, which were consistent with the cluster name and pattern reported in a study conducted by Kang et al. [18] In addition, studies with the same cluster name and pattern [15] and studies with similar cluster patterns but different cluster names [17] were also identified. *Psychosis symptoms* consisted of hallucinations, anxiety, elation/euphoria, delusions, and depression/dysphoria. Except for depression/dysphoria, the remaining symptoms were found to be symptoms included in the *psychosis symptom* cluster identified in previous studies [15–18, 43]. However, in these studies, depression was also classified as *mood* [17, 43], *emotional* [16], and *affective clusters* [15, 18]. Moreover, in other studies, euphoria has not been grouped together with other symptoms but has been classified as a single symptom cluster [15, 17]. *Physical behaviour symptoms* consisted of appetite and eating abnormalities, apathy/indifference, aberrant motor behaviour, and sleep and night-time behaviour disturbances. A few previous studies [17, 18, 43] reported similar cluster patterns with different cluster names than those of this study, while other studies have reported that aberrant motor behaviour was classified as a common symptom belonging to a symptom cluster of the same name “physical behaviour symptoms.” [16, 17] The diversity and variation in cluster classifications and nomenclature, with varying agreements, similarities, and differences between studies, are thought to be due to differences in the characteristics of the subjects, sample size, and analytical methods. While this study found correlations between each related symptom, further studies are required to confirm its BPSD-symptom-cluster classification, determine generalised BPSD clusters, and propose intervention programmes through cluster-specific approaches.

Testing the model of this study showed that the three symptom clusters had a direct effect on the family caregiver burden, whereby the *psychosis symptoms* cluster was the most influential. The results also found that the stages of dementia, ADL, and IADL indirectly affected family caregiver burden through hyperactivity, psychosis, and physical behaviour symptoms, indicating that BPSD had a total mediating effect. Garre-Olmo et al. [44] reported that BPSD in patients with AD had a direct effect on caregiver burden and ADL had an indirect effect on caregiver burden through BPSD, which was consistent with the results of this study. In addition, a study by Onishi et al. [45] showed that the severity of dementia and physical disability had a direct effect on caregiver burden, which was similar to the results of this study. In the modified model of this study, cognitive function demonstrated no effect on BPSD and caregiver burden. In the study conducted by Kang et al. [41], cognitive impairment status, sub-domains of neuropsychological test’s memory function, and frontal executive function showed correlation with caregiver burden measured using K-MMSE. The results are similar to those that did not act as an influence factor in the regression analysis. However, in the meta-analysis for family caregivers of home-dwelling elderly people with dementia [46], the factors such as family caregiver’s characteristics, problematic behaviour, cognition (memory), stages of dementia, and ADL have an effect on the caregiver burden. In particular, ADL [47, 48] and IADL [48] are also factors that affect the QOL for dementia patients. Therefore, when providing interventions for caregiver burden due to BPSD in patients with AD, it is necessary to explore strategies and algorithms that consider the different stages of dementia to be able to develop the necessary interventions, while focusing on slowing disease progression and improving functional independence in daily living. Furthermore, repeated studies are needed to determine whether cognition acts as an influencing factor on caregivers’ burden by utilising various scales that are used to measure cognition.

In accordance with the “Global action plan on the public health response to dementia (2017–2025)” announced by the World Health Organization [49], the Dementia Friendly Communities emerged, supported by the government’s Dementia Management policy. The Korean Ministry of Health and Welfare has proposed a dementia system, and they plan to institute dementia care centres in villages as a national imperative, which is part of its continual expansion strategy [50]. However, this is a long-term and complex project that requires appropriate preparation, budget allocations, and consensus among community members. It is, therefore, recommended that the government authorities fully support the development and establishment of systematic strategies to expand programmes and actively publicise them, so that patients with dementia and their family caregivers can utilise them.

Since patients with dementia are in chronic and disabling conditions and the family caregivers can be seen as a hidden patient [51], strategies to reduce caregivers’ burden due to BPSD and improve caregivers’ QOL should also be considered. In a previous study [52], if the caregiver burden was high, the occurrence of AD in patients with neuropsychiatric symptoms (NPS) was more frequent and severe. Engagement coping strategies of the caregiver showed a mediating effect between caregiver burden and NPS. Another study [53] that measured coping strategies of the AD patient’s caregiver utilised the Coping Inventory for Stressful Situations (CISS). This study reported that the success of the caregiver is related to task-focused and avoidance-focused strategies. The results of the other study showed that emotion-focused coping strategies decreased BPSD and caregiver burnout [54]. Heavy burden and stress among caregivers who take care of dementia patients can lead to poor QOL of AD patients [55]. A correlation was observed between caregiver burden and QOL [56] because BPSD and caregivers’ burden have an effect on the QOL of the caregiver [48, 57]; adequate coping strategies for reducing the burden and stress are required for improving the QOL of the caregiver [58]. In particular, Korean dementia families tend to use passive attitudes, avoidance, and anger [59]; therefore, they need an active intervention to train their families’ effective problem-oriented coping.

Although there is a need for customised intervention according to the preference of patients and their family caregiver, in Korea, it is difficult to apply a person-centred care model that individually supports the target, as it is based on specialised medical models such as doctors and social workers. In addition, the institutional foundation for universal welfare based on the social model is not yet firmly established, as the cost of health and social services is eased, but it remains an economic burden to dementia patients and their family members. Therefore, it is necessary to decide the direction of dementia policy through philosophical discussion in Korea, deviating from simply comparing and applying dementia policy cases in other countries [60]. Discussion on person-centred dementia care has recently expanded [61, 62] to ensure that dementia patients and their family caregivers are the main agents of the policy.

The findings of this study may not be applicable to the general population as the participants in this study were outpatients. Thus, these findings are limited in terms of generalisation and interpretation. In addition, this study used data previously collected for diagnostic and therapeutic purposes and was limited in describing family caregivers’ burden using a multidimensional approach that included characteristics, such as social position, monthly income, work position, caregiving time demands, home care service costs, medical device costs, educational level, and psychological factors [63–65]. Longitudinal studies covering the time span of disease progression, commencing from the time of the diagnosis of dementia, are recommended to determine changes in the factors affecting the classification of BPSD and family caregivers’ burden as the disease progresses. Finally, it is necessary to analyse how the coping strategies of the caregiver medicate the BPSD symptom clusters, the caregiver burden, and the QOL of the patient and family caregiver while controlling the general characteristics of the patient and the caregiver, such as sex, age, and education level.

## Conclusions

This study confirmed that the stages of dementia, ADL, and IADL affected BPSD and that BPSD, in turn, affected family caregivers’ burden; therefore, this study provides a basis for broadening the understanding of family caregivers’ burden. The results of this study are important, as they provide a strong foundation for future studies on multidimensional approaches to determine the relationship between BPSD in patients with AD and their family caregivers’ burden. These results can also be used for the development of treatment and nursing strategies for AD patients in clinical settings and can further contribute to policy development.

## Data Availability

The datasets generated and analysed during the current study are not publicly available due [personally sensitive medical records] but are available from the corresponding author on reasonable request.

## Abbreviations

AD: Alzheimer’s disease
BPSD: Behavioural and psychological symptoms of dementia
ADL: Activities of daily living
IADL: Instrumental activities of daily living
EMR: Electronic medical records
CRCD: Clinical Research Center for Dementia
K-MMSE: Korean version of the Mini-Mental State Examination
CDR: Clinical Dementia Rating
SGDS-K: Short form of Geriatric Depression Scale-Korea
K-BADL: Korean version of the Barthel ADL Index
S-IADL: Seoul-instrumental activities of daily living
K-NPI: Korean Neuropsychiatric Inventory
KMO: Kaiser–Meyer–Olkin
GFI: Goodness-of-Fit Index
AGFI: Adjusted Goodness-of-Fit Index
CFI: Comparative Fit Index
NFI: Normed Fit Index
IFI: Incremental Fit Index
SRMR: Standardized Root Mean Square Residual
RMSEA: Root Mean Square Error of Approximation
